# Editorial: Viral escape of mucosal immunity in sexually transmitted diseases

**DOI:** 10.3389/fimmu.2023.1273515

**Published:** 2023-08-23

**Authors:** Elena Criscuolo, Francesca Pala, Gabriel Siracusano, Assunta Venuti

**Affiliations:** ^1^ Laboratory of Microbiology and Virology, Vita-Salute San Raffaele University, Milan, Italy; ^2^ Laboratory of Clinical Immunology and Microbiology, National Institute of Allergy and Infectious Diseases, National Institutes of Health, Bethesda, MD, United States; ^3^ Division of Immunology, Transplantation and Infectious Diseases, San Raffaele Scientific Institute, Milan, Italy; ^4^ International Agency for Research on Cancer (IARC), World Health Organization, Lyon, France

**Keywords:** STD (sexually transmitted diseases), HIV - human immunodeficiency virus, mucosae, microbioma, therapy, vaccine

The mucosae found in both male and female genital tracts are the main entrances for sexually transmitted diseases (STDs). These diseases can come from a variety of sources such as viruses, bacteria, fungi, and parasites. Annually, there are approximately 2.6 million reported cases of STDs (1). This route of infection has seen an increasing number of new cases, especially among women who are more likely to contract STDs than men (2). It is essential to trigger both innate and adaptive immune responses at the entry points of these pathogens to prevent infection. However, co-evolving with the host, viruses have evolved strategies to counteract the immune control, promoting their survival and persistence. It is central to comprehend the mucosal factors that protect against sexually transmitted pathogens. These factors include humoral immune responses, antibody neutralization, non-neutralizing related functions, immune cell activation, and the role of microbiota in mucosal responses. This understanding can help reduce the risk of infection and offer new possibilities for effective vaccines and microbicide strategies, which have seen varying levels of success so far. (3, 4).

Mucosal surfaces are highly susceptible to infections but have a diverse range of innate and adaptive mechanisms to combat pathogens. The likelihood of a virus causing an infection depends on various factors related to the host. These factors may involve the mucosa’s physical barrier, the number of target cells available, changes in the microbiota of the mucosa, the humoral response, and immune activation caused by genital inflammation resulting from other sexually transmitted infections. The risk of transmission is linked to specific obstacles within the host, leading to a selection bias that benefits viruses with a higher potential for transmission between hosts.

There is still a lack of comprehensive knowledge regarding how viruses interact with the host at these specific anatomical sites. The aim of this Research Topic is to shed light on the critical interactions of viruses with the immune system of the genital mucosa that allow the virus to escape from it and eventually complete its life cycle.

A review by Caputo et al. examined how HIV interacts with the innate response of mucosal tissue. The various mucosae serve as a protective barrier that can be weakened by the infection, and the interaction between the virus and cells triggers an immune response that can help to contain the spread of the virus. When it comes to mucosal transmission of HIV, the varied histological structures and innate immunological components present in different mucosal surfaces can impact the effectiveness of transmission. Weakening of mucosal integrity can make infection easier, but a pro-inflammatory environment may actually offer some protection against the virus. Extensive research is being conducted on vaccination strategies against oral and other mucosal HIV transmissions. However, progress towards an effective vaccine is hindered by the lack of consensus on immune correlates of protection, as well as the absence of effective mucosal adjuvants and delivery systems. A strategic approach to blocking the replication of HIV and preventing its spread and dissemination is to contain it immunologically in the mucosa.

Additionally, it’s worth noting that genital fluids contain proteins that can actually increase the risk of viral infection. This includes semen-derived enhancers of virus infectivity and complement. The research by Baratella et al. investigates the early stages of HIV entry via the colonic mucosa. Specifically, they examined the impact of human seminal plasma on the intestinal permeability by analyzing the integrity of tight and adherent junctions. Additionally, they explored whether the presence of seminal plasma encourages the recruitment of MNP cells to the apical side of the human colonic mucosa. While there were noticeable variations in cytokine and chemokine levels between individuals with and without HIV, there was no impact on tissue morphology. However, it was noticed that a certain group of MNPs, which expresses CD11c, migrated towards the apical side of the colonic mucosa. This movement acts as a way for the virus to come into close proximity with the colonic mucosa and the associated cells.

Moreover, the impact of the mucosal microbiota on the facilitation or prevention of HIV infection is gaining particular attention from scientific research. The review by Caputo et al. discusses the potential impact of changes in the microbiome on the development of opportunistic infections. It also proposes that modulating the microbiome could potentially protect the host. The research by Ackerley et al. describes the distinct T cell compartment and microbiome composition found in the rectal environment of young men who have sex with men (YMSM) when compared to older males. In YMSM, there were lower levels of CCR5 expression in CD4+ T cells, increased CD4+ T cell proliferation, low levels of particular subsets of pro-inflammatory cytokine-producing CD4+ and CD8+ T cells. The researchers found that specific anaerobic genera, such as *Prevotella, Peptoniphilus, Lawsonella*, and *Anaerococccus*, were more prevalent in the microbiome of YMSM. Upon further analysis, they discovered that a higher abundance of these particular variants was associated with increased HIV viral replication in the explant model. While the study did not directly link the composition of the gut microbiome to rectal mucosa immune cell subsets, the researchers hypothesize that it may impact other aspects of the rectal immune environment, such as innate immunity, adaptive immune cells functionality, and production of inflammatory markers.

Last, the development of novel therapeutic strategies may address the mucosal surfaces. Fisher et al. highlight that although there have been multiple biomedical prevention methods introduced for women, such as topical tenofovir gel as pre-exposure prophylaxis (PrEP), or intra-vaginal rings with antiretroviral drugs, the levels of protection provided have been inconsistent. In their study, prior topical PrEP did not modulate envelope-specific IgG Fc mediated antibody-dependent cellular cytotoxicity (ADCC), and they suggest that the combination of PrEP use and vaccination eliciting these antibodies may serve as an HIV prevention strategy. The anti-envelope antibodies did not prevent infection in the study, but anti-V1V2-gp70, gp120, and p24 which trigger ADCC can be linked to slower disease progression (5), elite control (6), and a functional immune response (7). There is a possibility that these antibody functions may be specific to certain compartments of the body to avoid immunological redundancies or extra inflammation associated with non-neutralizing antibody-mediated activities. Overall, the findings indicate that non-neutralizing antibodies play a vital role in developing a varied and effective immune system, especially in the female reproductive tract.

We hope that this Research Topic will stimulate more research and clinical trials to better understand the capability of viruses to escape the mucosal immune system in sexually transmitted diseases.

## Author contributions

EC: Writing – original draft. FP: Writing – review & editing. GS: Writing – review & editing. AV: Writing – review & editing.
